# Supplementary figures and images for: Albendazole specifically disrupts microtubules and protein turnover in the tegument of the cestode Mesocestoides corti
Source: PLoS Pathog. 2025 Jun 4;21(6):e1013221. doi: 10.1371/journal.ppat.1013221 (PMC12162102; doi:10.1371/journal.ppat.1013221)

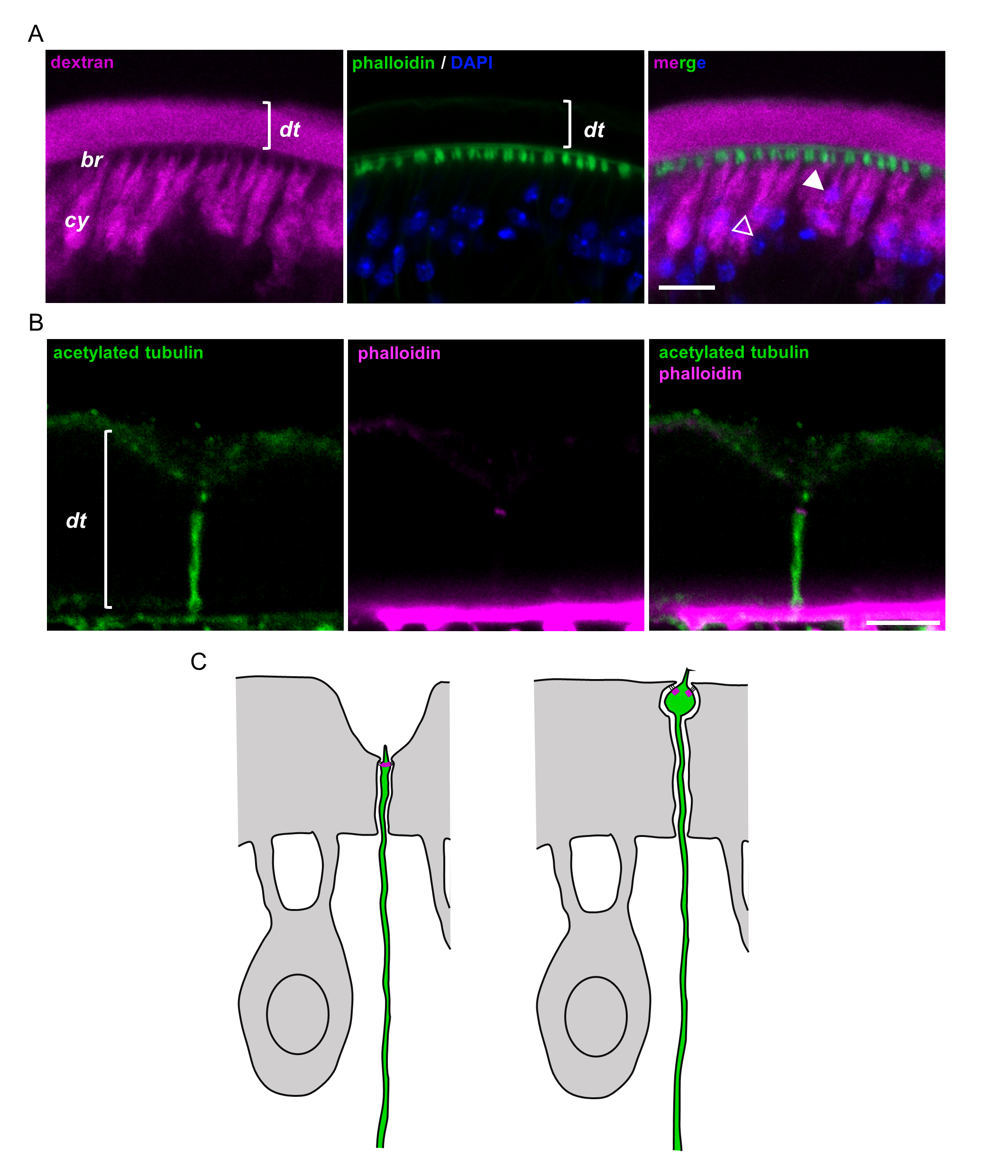

Supplement: S1 Fig — (A) Co-labeling of the tegument and muscle fibers with dextran and phalloidin, respectively, showing how cytoplasmic bridges traverse the outer muscle layer in between the muscle fibers, just below the distal tegument. Not all cells in the subtegumental region are tegumental cytons: the filled arrowhead indicates a dextran+ cyton, and the open arrowhead indicates a dextran- nucleus. (B) We found two distinctive morphologies of putative sensory nerve endings, both of which appear to end in a cilium-like protrusion. One sensory receptor has a dilated apical region (shown in Fig 2B in the main text) while the other, shown here by immunofluorescence for acetylated tubulin, does not have a dilation and appears to be surrounded by a depressed conical region of the distal tegument. (C) Schematic drawings of the two sensory nerve ending morphologies traversing the tegument. The possible position of septate junctions, based on phalloidin staining and previous electron microscopy descriptions are represented in purple, with black lines. dt, distal tegument; br, cytoplasmic bridges; cy, cytons; dt, distal tegument. Scale bars: A: 10 μm; B: 5 μm. (TIF) [file ppat.1013221.s001.TIF]

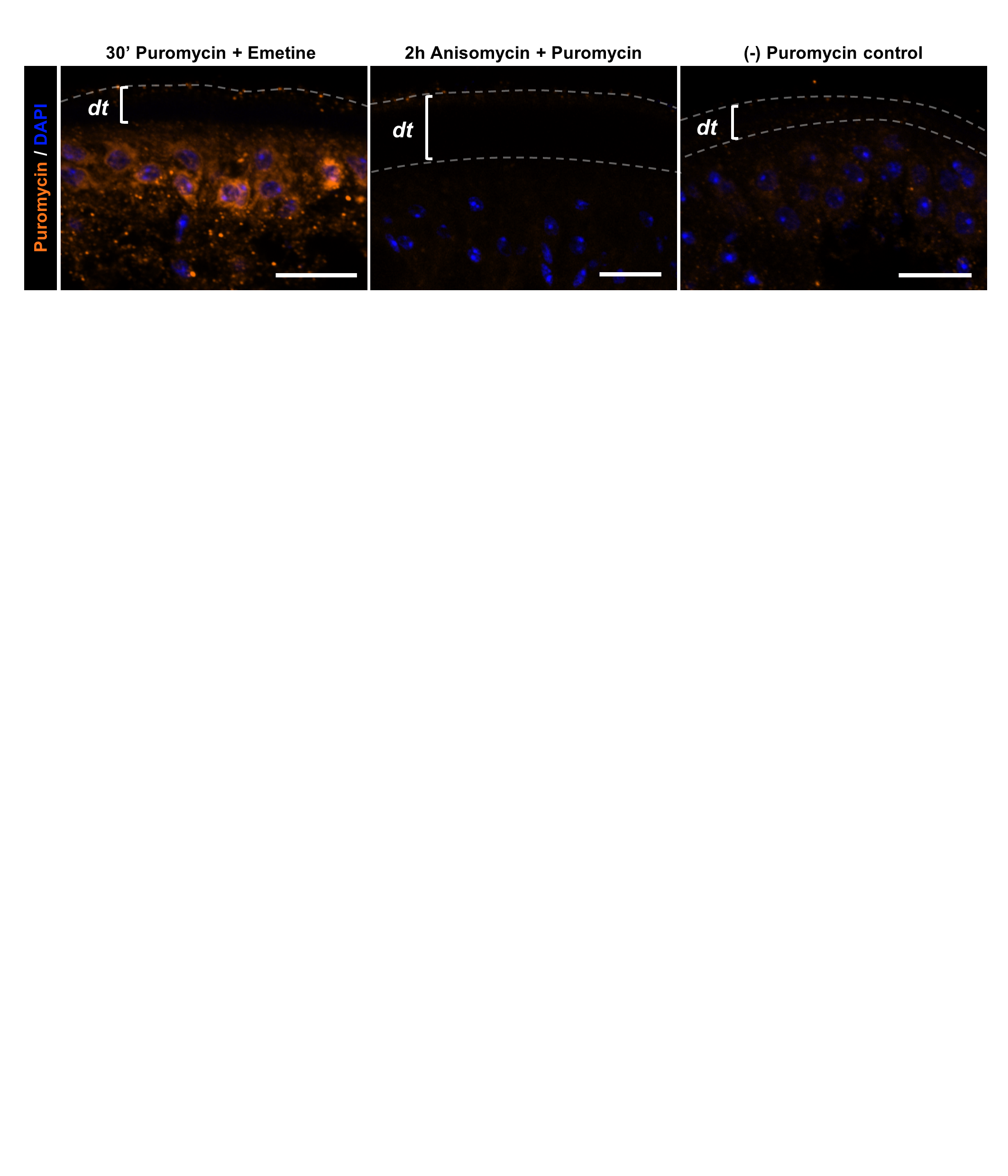

Supplement: S2 Fig — Emetine prevents the release of the puromycin labeled peptide from the ribosome, ensuring the observation of the protein synthesis sites (left panel). We obtained essentially identical results with and without emetine. A specificity control with anisomycin (which inhibits protein translation; middle panel) is shown, as well a control without puromycin (right panel). Scale bars: 10 μm. (TIF) [file ppat.1013221.s002.TIF]

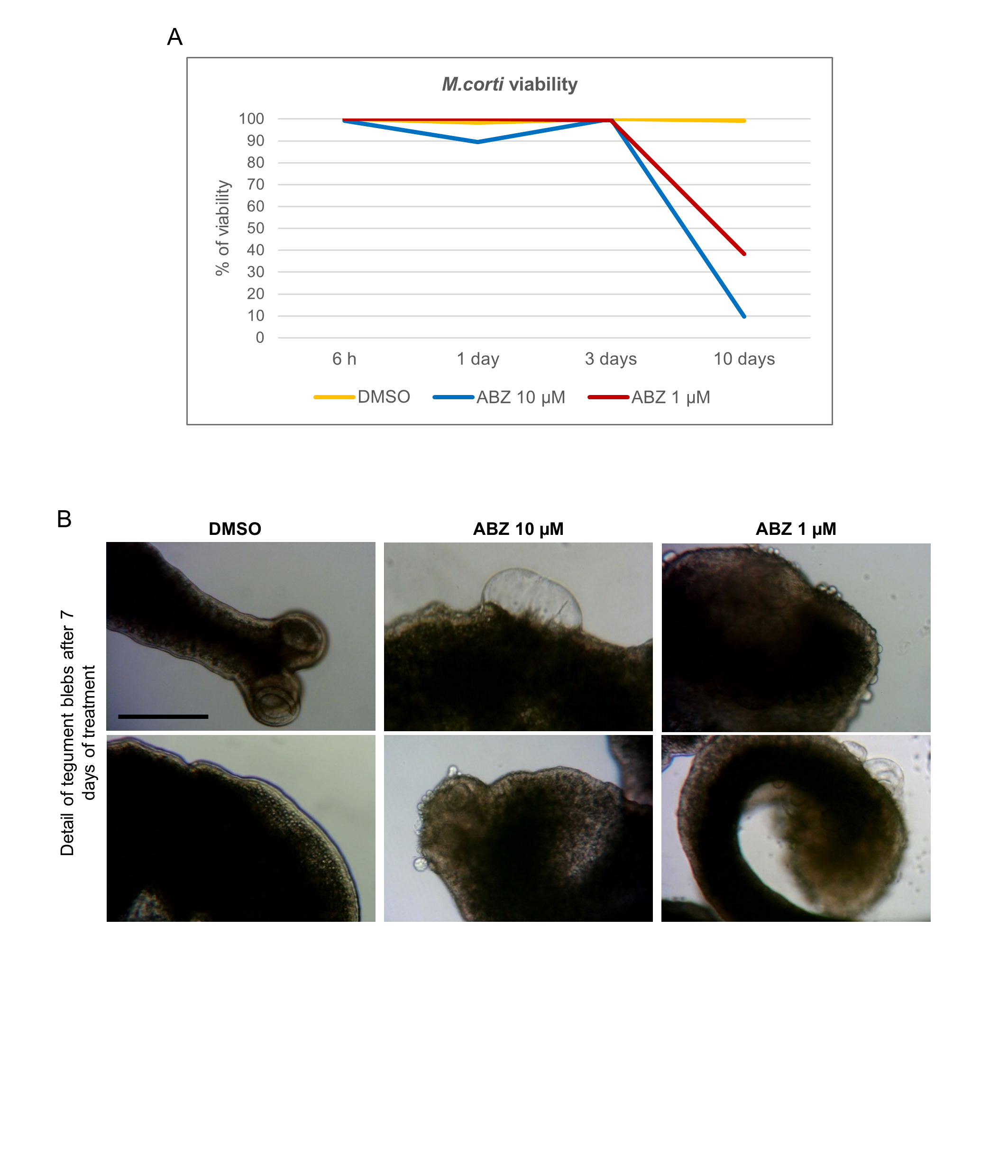

Supplement: S3 Fig — (A) Parasites were cultured in vitro in the presence of ABZ 1 µM or 10 µM. Viability was assessed by the eosin exclusion method. Viability was not significantly affected for at least 3 days, and then decreased to 38% and 10% for ABZ 1 µM and 10 µM, respectively, by day 10. (B) Detailed pictures of live parasites show blebs evidencing damage in the tegument of ABZ treated parasites on day 7. Scale bar: 200 μm. (TIF) [file ppat.1013221.s003.TIF]

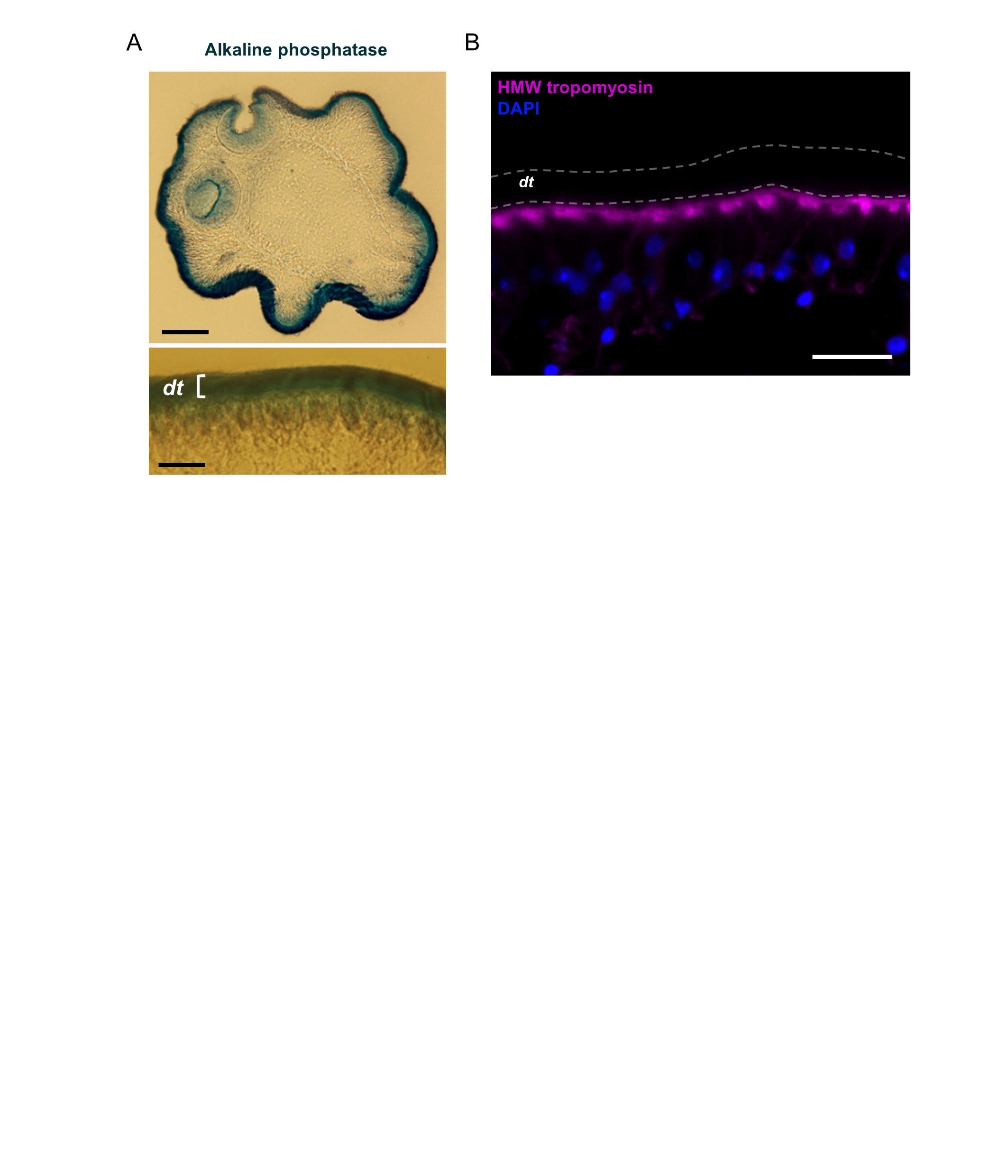

Supplement: S4 Fig — (A) Localization of the enzyme alkaline phosphatase revealed by histochemistry on cryosections shows that it is mainly located in the distal tegument. (B) Detection of high molecular weight (HMW) tropomyosins show their location immediately below the distal tegument. dt, distal tegument. Scale bars: upper A: 100 μm; lower A and B: 10 μm. (TIF) [file ppat.1013221.s004.TIF]

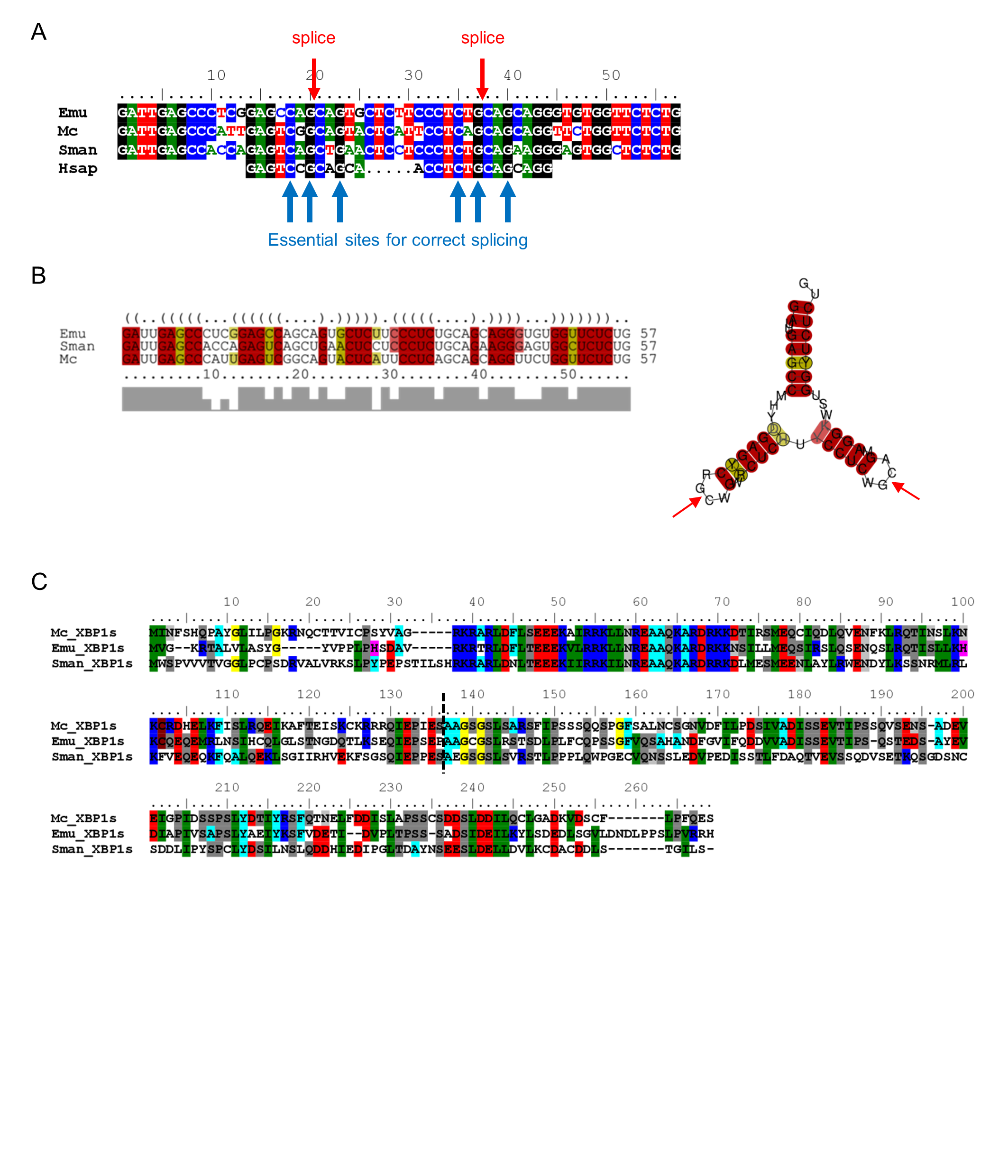

Supplement: S5 Fig — (A) Alignment of the region of XBP1 transcripts of Echinococcus multilocularis (Emu), Schistosoma mansoni (Sman) and M. corti (Mc) surrounding the 17 bp non-canonical intron (+/- 20 bp), labeled according to sequence conservation. The corresponding sequence of Homo sapiens XBP-1 is shown for comparison, including those residues that are essential for non-canonical splicing. (B) Structural prediction of the region of XBP1 transcripts from parasitic flatworms surrounding the non-canonical intron. The alignment in the left shows the conservation of the predicted secondary structure, which is shown on the right. The secondary structure consists of a double stem-loop structure similar to the unconventional splice junction of other species. (C) Protein sequence alignment of the spliced form of XBP1 (XBP1s) from Mc, Emu and Sman showing conservation at the amino acid level. Black dotted line marks the position of the frameshift produced by non-canonical splicing. (TIF) [file ppat.1013221.s005.TIF]

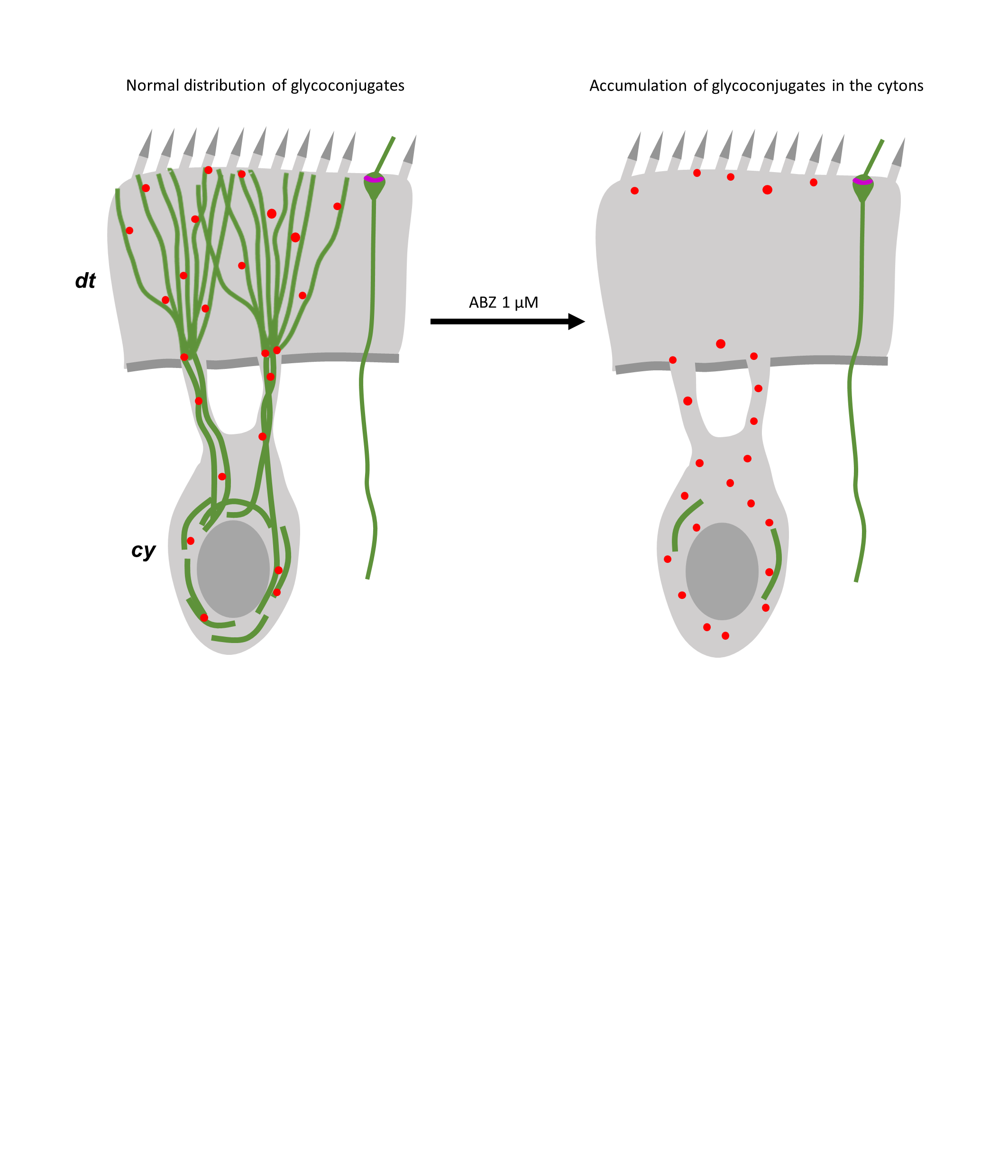

Supplement: S6 Fig — The schematic drawing depicts the effects on the tegument of treatment with ABZ: disappearance of microtubules in the distal tegument, and a concomitant accumulation of secretory material in the cytons. dt, distal tegument; cy, cytons. (TIF) [file ppat.1013221.s006.TIF]

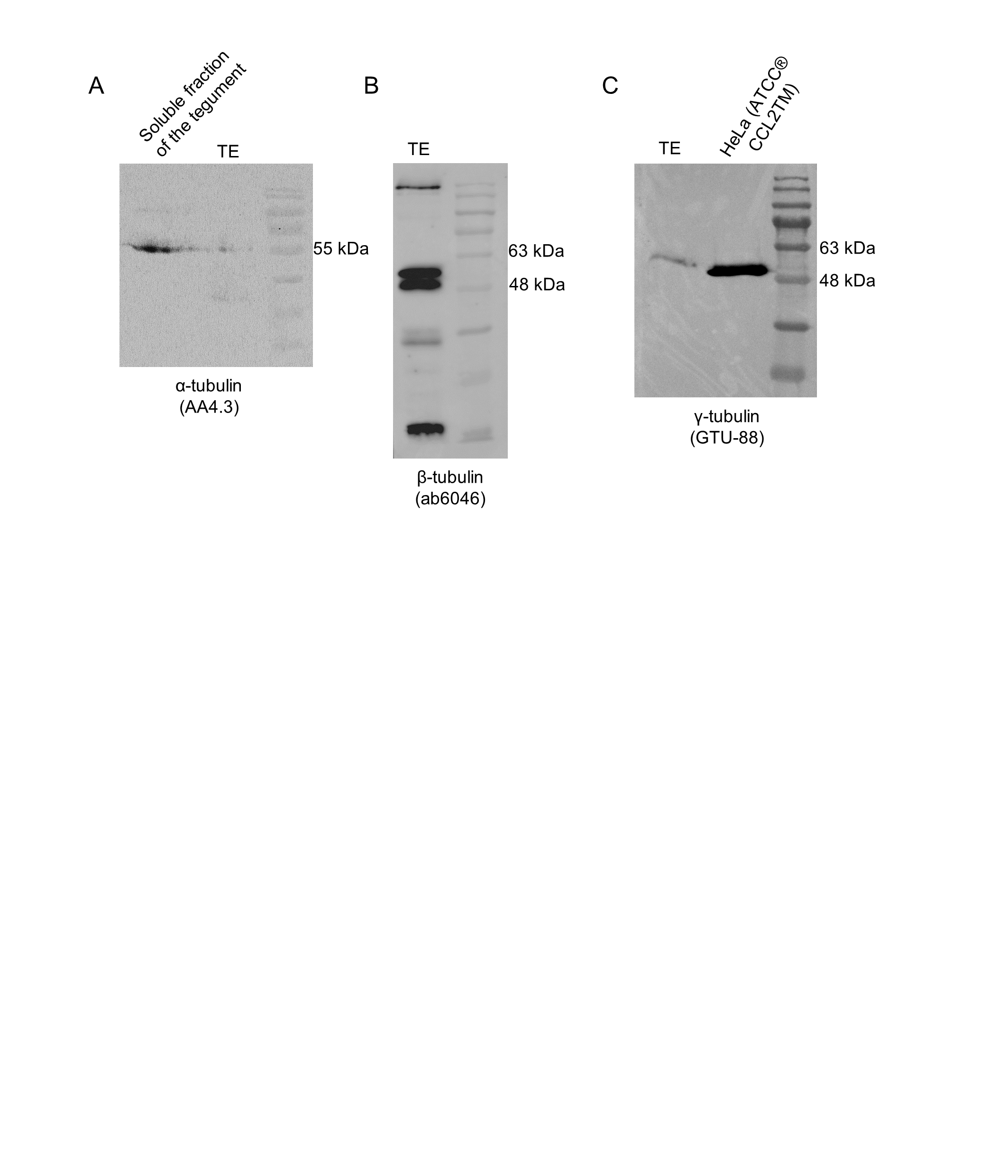

Supplement: S7 Fig — (A) Detection of alpha-tubulin shows a unique band of appropriate size in both soluble tegument fraction and total extracts (TE) of M. corti. (B) Detection of beta-tubulin shows two main bands in the middle range, the upper one has the expected size and the band immediately below could correspond to partial degradation of beta-tubulin in the extracts. (C) Detection of gamma-tubulin in total extracts of M. corti resulted in a single band of appropriate size. Total extract of HeLa cells was used as a positive control. (TIF) [file ppat.1013221.s007.TIF]
