# Supplementary material for: Albendazole specifically disrupts microtubules and protein turnover in the tegument of the cestode Mesocestoides corti
Source: PLoS Pathog. 2025 Jun 4;21(6):e1013221. doi: 10.1371/journal.ppat.1013221 (PMC12162102; doi:10.1371/journal.ppat.1013221)
Supplement: S1 Table — (PDF) [file ppat.1013221.s008.pdf]

Supplementary Table 1. Antibodies and their dilutions				
**Primary antibodies**	**Dilution for IHF**	**Dilution for Western Blot**	**Original epitope**	***M. corti* epitope**
AA4.3 (DSHB, U.S.A.; anti-α-tubulin, mouse monoclonal, culture supernatant. RRID: AB_579793)	1 in 10	1 in 200		
GTU-88 (Sigma, T5326, Israel; anti-γ-tubulin, mouse monoclonal. RRID: AB_532292)	1 in 250	1 in 1000	EEFATEGTDRKDVFFY	EEFATNGSDRKDVFFY
ab6046 (Abcam, U.S.A.; anti-β-tubulin, rabbit polyclonal. RRID: AB_2210370)	1 in 200	1 in 500		
6-11B-1 (Sigma, T7451, U.S.A.; anti-acetylated tubulin, mouse monoclonal. RRID: AB_609894)	1 in 500	-		
Y10b (DSHB, U.S.A.; anti-rRNA, mouse monoclonal, culture supernatant. RRID: AB_2313703)	1 in 3	-		
12D10 (Millipore, MABE343, U.S.A; anti-puromycin, mouse monoclonal. RRID: AB_2566826)	1 in 500	-		
Anti-High Molecular Weight Tropomyosins (Koziol et al 2011; rabbit polyclonal).	1 in 500	1 in 1000		
Anti-McU_009411 (GeneScript; anti-Alkaline Phosphatase, rabbit polyclonal against LPEVDLPQPPYPIKC-KLH)	-	1 in 1000		
Anti-Tyrosinated-tubulin (Thermo, MA1-80017, United Kingdom; rat monoclonal. RRID: AB_2210201)	1 in 100	-		
Anti-Phospho-eIF2α (Invitrogen, 44-728G, India; rabbit polyclonal. RRID: AB_2533736)	-	1 in 1000	SELSRRRIRSINK	SELSRRRIRSINK
**Secondary antibodies**				
Anti-Mouse-Alexa 555 Highly cross-adsorbed (Thermo, A21424, U.S.A. RRID: AB_141780)	1 in 500	-		
Anti-Rabbit-Alexa 546 (Thermo, A11010, U.S.A. RRID: AB_2534077)	1 in 1000	-		
Anti-Rat-Alexa 488 Highly cross-adsorbed (Thermo, A48262, U.S.A. RRID: AB_2896330)	1 in 200	-		
Anti-Mouse-HRP (Thermo, A16084, U.S.A. RRID: AB_2534758)	-	1 in 2000		
Anti-Rabbit-HRP (Thermo, A16104, U.S.A. RRID: AB_2534776)	-	1 in 2000		
