# Supplementary material for: Albendazole specifically disrupts microtubules and protein turnover in the tegument of the cestode Mesocestoides corti
Source: PLoS Pathog. 2025 Jun 4;21(6):e1013221. doi: 10.1371/journal.ppat.1013221 (PMC12162102; doi:10.1371/journal.ppat.1013221)
Supplement: S2 Data — (PDF) [file ppat.1013221.s010.pdf]

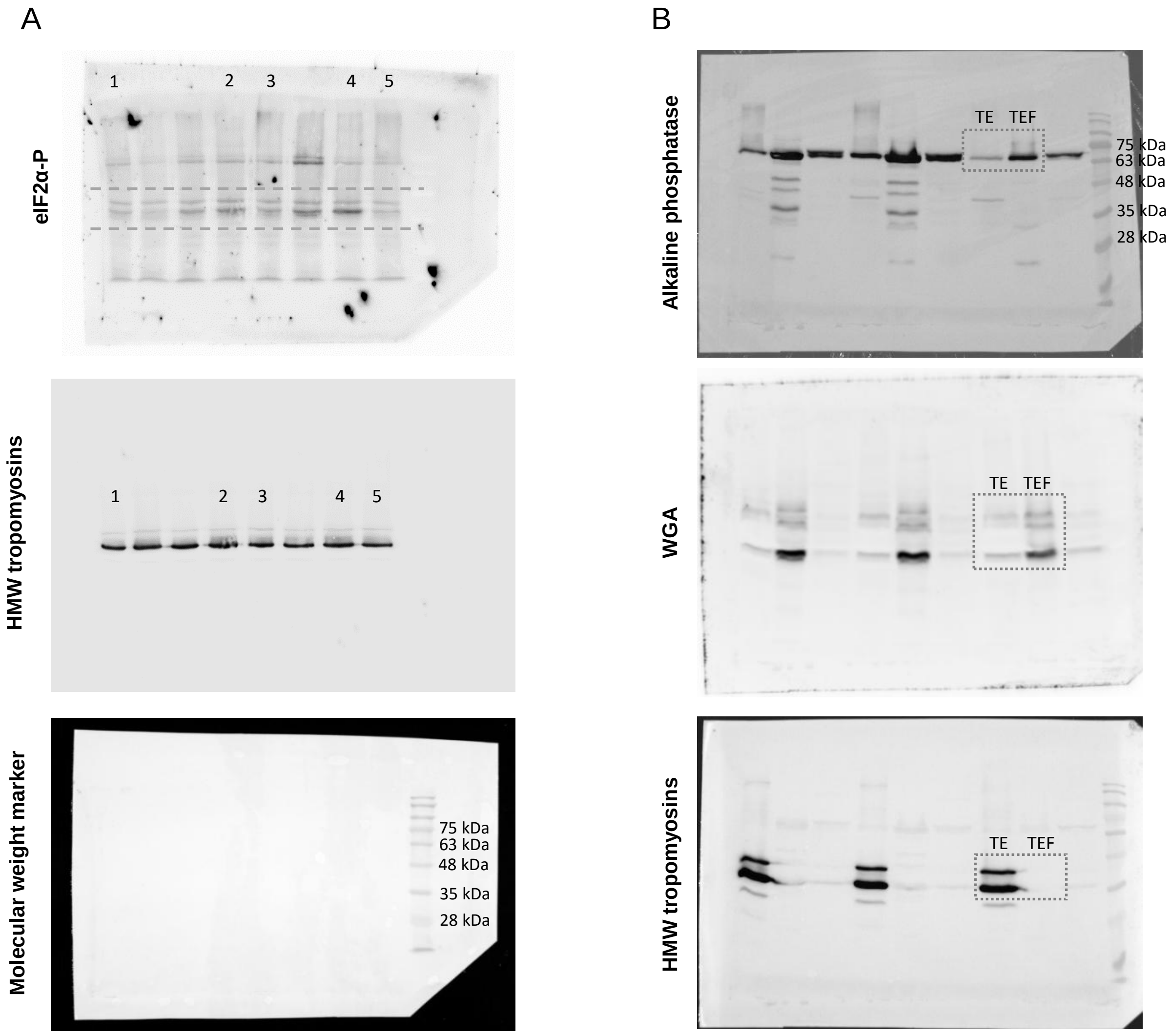

Supplementary Data 2. Original Western Blot Images (A) Western blot to detect eIF2α-P (upper panel). Dotted gray lines show where the bands were cropped for Fig. 7A. Lanes: 1-DMSO, 2-ABZ 10 µM, 3-ABZ 1 µM, 4-DTT 1 mM, 5-No treatment. Middle panel shows detection of high molecular weight (HMW) tropomyosins, used for normalization. Lower panel shows the molecular weight marker. (B) Western blot to detect Alkaline phosphatase in Total Extracts (TE) and Tegument Enriched Fractions (TEF) (upper panel). Dotted gray square indicates where the bands were cropped for Fig. 5D. Middle panel shows glycoconjugates detected with WGA on the same blot as A, and lower panel shows HMW tropomyosins, again, on the same blot as A.
